# Rigor and reproducibility of hollow-fiber infection model for antiviral pharmacokinetics/pharmacodynamics studies

**DOI:** 10.1128/aac.00969-26

**Published:** 2026-09-03

**Authors:** Shashikant Srivastava, Tawanda Gumbo

**Affiliations:** 1 Division of Infectious Diseases, Department of Medicine, School of Medicine, University of Texas at Tyler 12347, Tyler, Texas, USA; 2 Department of Cellular and Molecular Biology, University of Texas Health Science Centre at Tyler, Tyler, Texas, USA; 3 IMPI Biotechnology Company Incorporated, Tateguru, Zimbabwe; Providence Portland Medical Center, Portland, Oregon, USA

**Keywords:** pharmacokinetics, pharmacodynamics, antiviral, drug, quality control

## LETTER

We read the recently published article by Sudarsono et al. (1) describing a hollow-fiber infection model (HFIM) for pharmacokinetic/pharmacodynamic (PK/PD) studies with antiviral drugs. The authors used cytomegalovirus (CMV), a heamodialyzer hollow-fiber cartridge, and ganciclovir to perform a PK/PD study to establish a CMV-HFIM that can be used for determining the PK/PD exposure target for optimal antiviral dose selection.

Use of the HFIM in antiviral drug development is not new, as illustrated and summarized elsewhere by Kembou-Ringert et al. (2). Since Sudarsono et al. (1) emphasized on developing a “low cost heamodialyzer hollow-fiber cartridge-based HFIM,” a few clarifications would help to strengthen their argument and further expand and promote the use of the HFIM model in a preclinical drug development space that, otherwise, is expensive and labor intensive. The authors listed the cost of the heamodialyzer hollow-fiber cartridge (dialysis cartridge, FX paed hemodialyzer, Fresenius Medical Care, Bad Homburg, Germany) as 25€. However, cartridge is only one component of the HFIM setup and running cost. As an example, the media inflow rate to establish the equilibrium between the central reservoir, extra-capillary space, and the intra-capillary space of the hollow fiber cartridge was set to 30 mL/min. In the simplest calculation, this means 43.2 L media per system per day, and when supplemented with 10% fetal bovine serum (FBS), as mentioned in the Methods section of the published article, will require 4.32 L FBS. This adds significant running cost and increases with the number of replicate units and longer study duration. Then, there are many other single-use components to assemble the HFIM; a comprehensive list is not appropriate to include in our commentary. Therefore, unless all these variables are considered and used to generate a cost estimate considering the length of the study and number of HFIM units used per experiment, the argument of “establishing a novel low-cost HFIM for antivirals” needs to be reevaluated.

On the experimental details, rigor and reproducibility, after the initial 3 days of growing two different strains of CMV using the human lung fibroblasts (MRC-5) cells and twice daily (q12h) of ganciclovir, the HFIM were sampled, and drug concentrations were measured for pharmacokinetic (PK) modeling and viral load estimation for pharmacodynamic (PD) analysis. First, the manuscript does not provide any data on MRC-5 cell viability over time, a crucial piece of information that would also be needed for intracellular drug concentration and exposure calculations, as suggested by the authors. Second, Figure 3 does not show the data points for the measured drug concentration for the second dosing of the q12h schedule. Third, by visual inspection of Figure 3, the *C*_max_ of intended 10 mg/L does not appear to be achieved, and the variance between replicate HFIM units needs to be reported. Fourth, Figure 4B shows the concentration–time profile for only 8 h, and Table 1 does not report the ganciclovir half-life in the HFIM, which was intended to be 4 h (3). Fifth, Table 1 reports AUC_12_ as 42.2 mg*h/L, whereas in Figure 3D, AUC_24_ is reported and appears to be the same value as for AUC_12_ by visually examining the *y*-axis. A clarification on these discrepancies will be useful. Since the authors report a novel HFIM for antiviral PK/PD, we believe the authors would agree that the bar for quality control and reporting should be set high for rigor and reproducibility as established in a number of regulatory and research publications for HFIM models developed for other infections (4–7).

In summary, while we agree with the authors that a fundamental understanding of the relationship between the drug exposure and viral dynamics is required for determining the clinically relevant and PK/PD optimized exposure targets for antiviral drugs (8, 9), more information is needed to establish the notion of a cost-effective heamodialyzer hollow-fiber cartridge-based HFIM for antiviral PK/PD.
